# Hydrogel capsules as new delivery system for *Trichoderma koningiopsis* Th003 to control *Rhizoctonia solani* in rice (*Oryza sativa*)

**DOI:** 10.1007/s11274-024-03897-0

**Published:** 2024-02-26

**Authors:** Mauricio Cruz-Barrera, Luisa Fernanda Izquierdo-García, Magda Gómez-Marroquín, Adriana Santos-Díaz, Liz Uribe-Gutiérrez, Carlos Andrés Moreno-Velandia

**Affiliations:** 1 Bioproducts Department, Corporación Colombiana de Investigación Agropecuaria (AGROSAVIA), Km 14 vía Bogotá a Mosquera, Mosquera, Colombia; 2 Agricultural Microbiology Laboratory, Tibaitatá Research Center, Corporación Colombiana de Investigación Agropecuaria (AGROSAVIA), Km 14 vía Bogotá a Mosquera, Mosquera, Colombia

**Keywords:** Biopolymer, Encapsulation, Desiccation tolerance, Formulation, Scaling-up

## Abstract

The incorporation of biological control agents (BCAs) such as *Trichoderma* spp. in agricultural systems favors the transition towards sustainable practices of plant nutrition and diseases control. Novel bioproducts for crop management are called to guarantee sustainable antagonism activity of BCAs and increase the acceptance of the farmers. The encapsulation in polymeric matrices play a prominent role for providing an effective carrier/protector and long-lasting bioproduct. This research aimed to study the influence of biopolymer in hydrogel capsules on survival and shelf-life of *T. koningiopsis*. Thus, two hydrogel capsules prototypes based on alginate (P1) and amidated pectin (P2), containing conidia of *T. koningiopsis* Th003 were formulated. Capsules were prepared by the ionic gelation method and calcium gluconate as crosslinker. Conidia releasing under different pH values of the medium, survival of conidia in drying capsules, storage stability, and biocontrol activity against rice sheath blight (*Rhizoctonia solani*) were studied. P2 prototype provided up to 98% survival to Th003 in fluid bed drying, faster conidia releasing at pH 5.8, storage stability greater than 6 months at 18 °C, and up to 67% of disease reduction. However, both biopolymers facilitate the antagonistic activity against *R. solani*, and therefore can be incorporated in novel hydrogel capsules-based biopreparations. This work incites to develop novel biopesticides-based formulations with potential to improve the delivery process in the target site and the protection of the active ingredient from the environmental factors.

**Supplementary Information:**

The online version contains supplementary material available at 10.1007/s11274-024-03897-0.

## Highlights

Hydrogel immobilization formulation approach for *T. koningiopsis*.

Enhancing drying survival of *T. koningiopsis* conidia.

Antagonist capacity of formulated *T. koningiopsis* against *R. solani* in rice.

Increasing storage stability of *T. koningiopsis* conidia.

## Introduction

The phytopathogen fungus *Rhizoctonia solani* Kühn (teleomorph *Thanatephorus cucumeris* Frank. Donk) has a broad spectrum of plant hosts, infects plants from around 32 families and 188 genera, and is considered a phytosanitary threath of global importance (Srinivasachary et al. 2011). The sheath blight disease caused by *R. solani* has been the second most damaging in rice crops in supplying countries (Sandoval and Cumagun 2019). The management of sheath blight is currently based on plant breeding and chemical fungicides, but other alternatives such as formulations based on beneficial microorganisms are also effective. Biocontrol fungi (BF) can reduce both the infections and the disease development in plants, through the lytic enzymes secretion, antibiotic compounds, and activation of the plant immune system (Srivastava et al. 2016). The control of *R. solani* is challenging due to the high survival of sclerotia into the soil, its highly extensive host range and its ecological performance (Nasimi et al. 2024). Many studies report the beneficial effects of the genus *Trichoderma* (Teleomorph: Hypocrea) as antagonist against *R. solani* (Melignani et al. 2023), as plant growth promoter as well as stimulator of plant defense responses (Fazeli-Nasab et al. 2022).

*Trichoderma* spp., are usually anaerobic, facultative, and cosmopolitan filamentous fungi that persist in several agricultural soils and in other substrates (Asad 2022; Sharma et al. 2022). Besides, the genus *Trichoderma* exhibits an exceptional range of lifestyles and interactions with *R. solani* indicating mycoparasitism as major biocontrol activity (Asad 2022; Melignani et al. 2023; Mukherjee et al. 2022). Particularly, the strain *T. koningiopsis* Th003 is a well characterized BF with rapid growth and low nutritional requirements, which makes it a good competitor and colonizer of the soil. This saprophytic fungus produces a great variety of metabolites, including cell wall-degrading enzymes such as cellulases, β-1,3 glucanases and chitinases (Hirpara et al. 2017; Sharma et al. 2022). Due to the production of these enzymes, *T. koningiopsis* Th003 can degrade sclerotia from phytopathogenic fungi such as *R. solani*, *Sclerotinia sclerotiorum* (Zapata et al. 2020) and *Sclerotium cepivorum* (Smith et al. 2013). Th003 is the active ingredient of the water-dispersible granules formulated bio-fungicide TRICOTEC® WG.

Diverse formulation approaches have been addressed to conserve and strengthen the biological performance of *Trichoderma* sp. conidia. Powders and granulates are the most common (He et al. 2019; Santos et al. 2012), that include the used of talc (Sriram et al. 2011), vermiculite, ethyl cellulose, biochar, peat, kaolin, chitosan, (Wijesinghe et al. 2011) among others. Another strategies involve microencapsulation techniques (Braga et al. 2019), invert emulsions (Batta 2004), seed coatings (Cortés-Rojas et al. 2021; Victoria Arellano et al. 2021), and liquid carriers (Herrera et al. 2020; Kumhar et al. 2020).There are more than 80 registered bioproducts based on *Trichoderma* to control *Rhizoctonia* in different crops worldwide, more than 70% of them are wettable powders (WP) (Woo et al. 2014). Nevertheless, there are still limitations regarding innovative formulates for improving preservation of biological activity, time for activation, number of applications, water demand, desiccation tolerance, the stability during storage and an effective targeting releasing of the active ingredient in the field, among others.

Considering the lack of cutting-edge formulates, the development of biopesticide formulations has led to new strategies, including the immobilization of microorganisms that appears promising for several functions (Humbert et al. 2017a). This approach involves the attachment or entrapment of cells in a polymeric matrix. Immobilization systems include surface adsorption, flocculation, covalent binding materials, cross-linking, and polymer encapsulation (Gong et al. 2022). Among these approaches, biopolymeric encapsulation is considered the most advantageous technique for the development of microorganism’s carriers (Szopa et al. 2022; Yerramathi et al. 2023). Therefore, encapsulated living cells are protected in a nutritious shell against mechanical and environmental stresses such as temperature, pH, organic solvents, predators, and others (Liu et al. 2023; Velloso et al. 2023). Once the biopolymeric-based capsules arrive to the soil, the native microbiota actively degrades them, facilitating the encapsulated cells to gradually release into the soil, regularly at rates according to periods of time for seed germination or the plant emergence (Cruz et al. 2020).

Hence, for positioning novel BF formulates such as biopolymeric capsules, the characterization of prototypes is mandatory to compare to the traditional formulates such as WG. Here, it was hypothesized that the biopolymer in capsules can influence the immobilized BF performance. Therefore, in this work two prototypes of hydrogel capsules containing conidia of *T. koningiopsis* Th003 were designed to efficiently control *R. solani* in rice. The influence of alginate and amidated pectin as main biopolymers on the physical-chemical and biological behavior of the prototypes was compared.

## Materials and methods

Alginate was acquired from Sigma Aldrich Corporation (Product number: W201502, Darmstadt, Germany) G/M ratio 1.05. Amidated pectin (Grinsted® LA 410, esterification degree 31%, amidation degree 19%) and calcium gluconate were provided by Cimpa (Bogotá, Colombia). All other materials were of analytical reagent grade and were used as received. *Plant material*: Rice seeds Fedearroz 2000 were provided by Fedearroz (Espinal, Colombia) as agrochemical-free seeds.

### Sources of fungal pathogen *Rhizoctonia solani *and biocontrol *T. koningiopsis* strain Th003

*T. koningiopsis* strain Th003 (GenBank AB568477) and *R. solani* strain Rh002 were supplied by the Microorganisms Germplasm Collection from Corporación Colombiana de Investigación Agropecuaria - AGROSAVIA RNC:129 - (Mosquera, Colombia). Rh002 was isolated from rice plants showing typical symptoms of sheath blight, and its pathogenicity on rice plants was previously tested under greenhouse conditions. Th003 was isolated from Andean soil and its biocontrol versatility has been previously demonstrated (Moreno-Velandia et al. 2018). Both strains were stored at -70 °C in sterile glycerol solution (10% w/v) and were reactivated and cultivated on potato-dextrose-agar plates (PDA, Oxoid®, Basingstoke, UK) at 27 ± 0.5 °C for seven days. Conidial suspension of Th003 was prepared in sterile Tween 80 solution (0.1% v/v) and the concentration was adjusted by counting in a hemocytometer (Barrera et al. 2019).

### Hydrogel capsules preparation

Two different hydrogel capsules were prepared by ionic gelation method (Cruz Barrera et al. 2020). The alginate and amidated pectin were dissolved in ultrapure water at 4.0%, maintaining constant stirring at 600 rpm and 70 ± 5 °C. Subsequently, either alginate or amidated pectin polymer matrix was prepared by mixing the fillers biochar vegetable origin (BioEspacio, Plantilandia, Colombia) and polydextrose (Cimpa, Colombia) for 15 min, adjusted to a final concentration as indicated in Table 1. Then, the conidial suspension of Th003 was added at a concentration of 2 × 10^7^ conidia mL^− 1^. After 15 min of stirring, the mixture was transferred to a 20 mL syringe and dripped through a cannula (diameter 2.1 × 0.8 mm, Sterican, B. Braun Melsungen AG, Melsungen, Germany) into the stirred crosslinking agent solution. Calcium gluconate was used as cross-linker at 0.1 M. Further, the capsules were separated from cross-linker solution using a sieve (mesh size: 1.0 mm) before drying operation.

**Table 1:** Hydrogel capsule prototypes made by immobilizing conidia of *T. koningiopsis* Th003

Polymeric matrix composition (p/v)	P1	P2
Alginate	3.0%	
Amidated pectin		3.0%
Polydextrose	3.7%	3.7%
Biochar	0.6%	0.6%
*T. koningiopsis* Th003 suspension	18.6%	18.6%
H_2_O	74.2%	74.2%

Calcium gluconate at 0.1 M as cross-linker solution. Drooping ratio 2:1, Cross-linker: Polymeric matrix

### Survival of encapsulated Th003 cells to drying process

A scalable fluid bed drying process was carried out to determine the effect of encapsulating biopolymer (alginate and amidated pectin) on the tolerance of Th003 conidia to desiccation conditions. Briefly, a fluid bed D-01277 (Glatt® GmbH, Binzen, Germany) was used, which was equipped with a chamber and inlet air temperature, pressure control, a flap to adjust the inlet air flow, and a spray air regulator. The air inlet temperature was settled at 37 °C, with the inlet flap set at 100%. For the drying process, the pressure drop was kept at a range value of 5 to 15 mBar. The capsules had an initial concentration of 1.0 to 2.0 × 10^6^ CFU/capsule, with moisture content > 98%. Drying batches of 500 g of wet capsules were prepared, with operating times at 60 min up to the final moisture content < 7%.

The viability of encapsulated Th003 conidia was assessed using the methodology outlined by Santos-Díaz et al. (2022) with some modifications. Before and after drying process, ten capsules were placed into a Erlenmeyer flask with 10 mL of citric acid (0.03 M) and sodium carbonate (0.05 M) sterile solution (pH 7 ± 2) for 1 h on a rotary shaker at 150 rpm and 15 °C. The IKA T25 ultra-turrax was operated for 5 min at 5000 rpm to maximize the rupture of the capsules. After complete dissolution, serial dilutions were plated onto PDA medium and incubated at 28 °C for 96 h and the number of colonies was counted.

### Efficacy of encapsulated prototypes against rice sheath blight

The encapsulated prototypes based on Th003, denotated as P1 and P2, were evaluated in a potted bioassay, in which rice plants were grown in soil artificially inoculated with *R. solani* Rh002, under greenhouse conditions. Plastic bags (0.5 × 0.75 m) were filled with soil (7 Kg) before the inoculation with Rh002 and were placed inside the pots. For pathogen inoculation, sterile broken rice grains and rice husk substrate (proportion 1:2, respectively), supplemented with Ko and Hora solution (Ko and Hora 1971), colonized by Rh002 was harvested and incorporated to 500 g of soil (4% w/w) from the top of the total potted soil. Rice seeds free of biocontrol agents and fungicides were sown (10 seeds per pot) and each planting site was treated with two capsules of each prototype. The effect of the prototypes was compared to the commercial biofungicide TRICOTEC® based on Th003, formulated as wettable dispersible granules – WG (1 × 10^9^ conidia g^− 1^). TRICOTEC (1 g L^− 1^) was applied by soil drenching 50 mL of suspension per pot once after planting (TRICOTEC-S) or following the complete manufacturer directions for use, i.e. by soil drenching at planting, one and three weeks after planting, and by spraying at 4 and 6 weeks after planting. (TRICOTEC-EC). The labeled as P11 and P12, were encapsulated controls featuring only the carriers from each formulation, without any presence of Th003.

The fungicide thifluzamide (Pulsor® 2SC, SummitAgro), at dose of 1.5 ml L^− 1^ sprayed on the plants at 30 and 45 days after planting, was included as a positive control. Rice plants grown in artificially inoculated soil with Rh002 without treatments against sheath blight were included as negative control; and plants grown in soil free of Rh002, without treatments against the disease, were included as absolute control. Once the plants emerged, approximately 20 days after planting, the soil was flooded continuously, and a plastic bag was placed upright to maintain high relative humidity in the foliar environment. The experiment was arranged in a randomized complete blocks design with three replications. The experimental unit consisted of one pot with 7 to 10 plants due to the emergence of seedlings not being uniform. The disease incidence, expressed as the proportion of symptomatic plants in each pot, and the disease severity were recorded on 14, 21, 28, 49, 62 and 69 days after planting. The disease severity was measured according to the scale described by Groth (2005) (Table 1S) and the disease intensity index was calculated through the equation 𝐼𝐼𝐸= ((Σ𝑆𝑖 ∗ 𝑁𝑖) ÷ (9 ∗ 𝑁) * 100, where *Si* is the severity level of the symptoms, *Ni* is the number of plants at each severity level *Si*, 9 is the maximum number of severity levels and *N* is the total number of plants in the experimental unit (Hervás et al. 1998).

### Study of prototypes stability under storage and mathematical estimation of shelf life

The stability test was carried out following the protocol described by Santos-Díaz et al. (2022). Briefly, 15 samples of 5 g of encapsulated prototypes P1 and P2 were packed in aluminum foil zipper bag and stored at three temperatures (8 ± 2, 18 ± 2 and 28 ± 2 °C). Before store and every two months the viability was determined as describe above. Experimental design was completely randomized with repeated measures over time. For estimate the shelf life, the viability (CFU) vs. storage time was correlated using the mathematical polynomial model, where *y* corresponds to the variable colony-forming units (CFU), *t* the storage time and *ki* to a constant.

$$Polynomial model:y=k1+k2t+k3{t}^{2}+k4{t}^{3}\dots.$$

The shelf life was defined as the time in which the viability was equal to or greater than 1.0 × 10^5^ CFU g^− 1^ (Santos et al. 2016; García et al. 2022; Santos et al. 2012).

### Determination of particle size

The particle size of capsules in P1 and P2 prototypes was determined by means of two perpendicular measurements of the diameter recorded to 100 capsules for each prototype. Data were obtained by light field optical microscopy through a Carl Zeiss stereomicroscope (Stemi 508, Germany), with adaptation of a micrometer at 10X magnification. A frequency diagrams, the sphericity factor (φ), and the average size of capsules were determined (Santos et al. 2022; Chan et al. 2011).

### Release profile of encapsulated conidia at different pH conditions

To understand the release rate of encapsulated Th003 conidia at different pH under in vitro conditions, the P1 and P2 prototypes were immersed in phosphate buffer adjusted at 5.8–7.4 pH simulating soil pH conditions of rice crops (Liu and Liu. 2009; Santos-Díaz et al. 2022).

### Scanning electron microscopy (SEM)

The surface morphology of the capsules was analyzed by scanning electron microscopy (SEM). Briefly, samples were fixed by immersion in 2% glutaraldehyde for 30 min and then washed with ethanol gradients (30%, 50%, 80% and 99%) for 15 min each (Cruz Barrera et al. 2020). At each step, the samples were dried in an extraction cabinet and placed in a desiccator with silica gel for 48 h as an additional dehydration step. Cross sections were cut from the capsules. The fixed and dried samples were coated with a 30 nm gold microfilm using a metallizer (Denton Vacuum Desk IV, LLC, USA). Observations were made using a JEOL JSM-6490LV microscope with the Evenhart-Thornley E-T detector (SEMTech Solutions InC, Massachusetts, USA).

### Statistical analysis

Data were analyzed using SPSS Statistics v.2 software (SPSS, Chicago, IL). Data were checked for normality and homogeneity of variances using the Shapiro-Wilk and Bartlett tests, respectively (α = 0.05). Data are presented as mean values ± standard deviations (SD) or standard error (SE). A multivariate analysis of variance (MANOVA) (Statgraphics Centurion XVII) was used to determine the effect on the conidia release by prototypes, pH, and time. Tukey’s honestly significant difference (HSD) test was used to discriminate differences among means of tretaments. For the bioassay under greenhouse, the area under the disease incidence and severity progress curves, and the efficacy of the treatments to reduce the progress of the disease incidence (AUDPC-INC) and severity (AUDPC-SEV) were subjected to one-way ANOVA. Data in percentage were transformed by the arcsine-square root transformation. The AUDPC was calculated by the equation described by Campbell and Madden (1990). Duncan multiple range test (α = 0.05) was carried out for means comparison between treatments.

## Results

### Survival of encapsulated conidia to the drying process

**Fig. 1:**
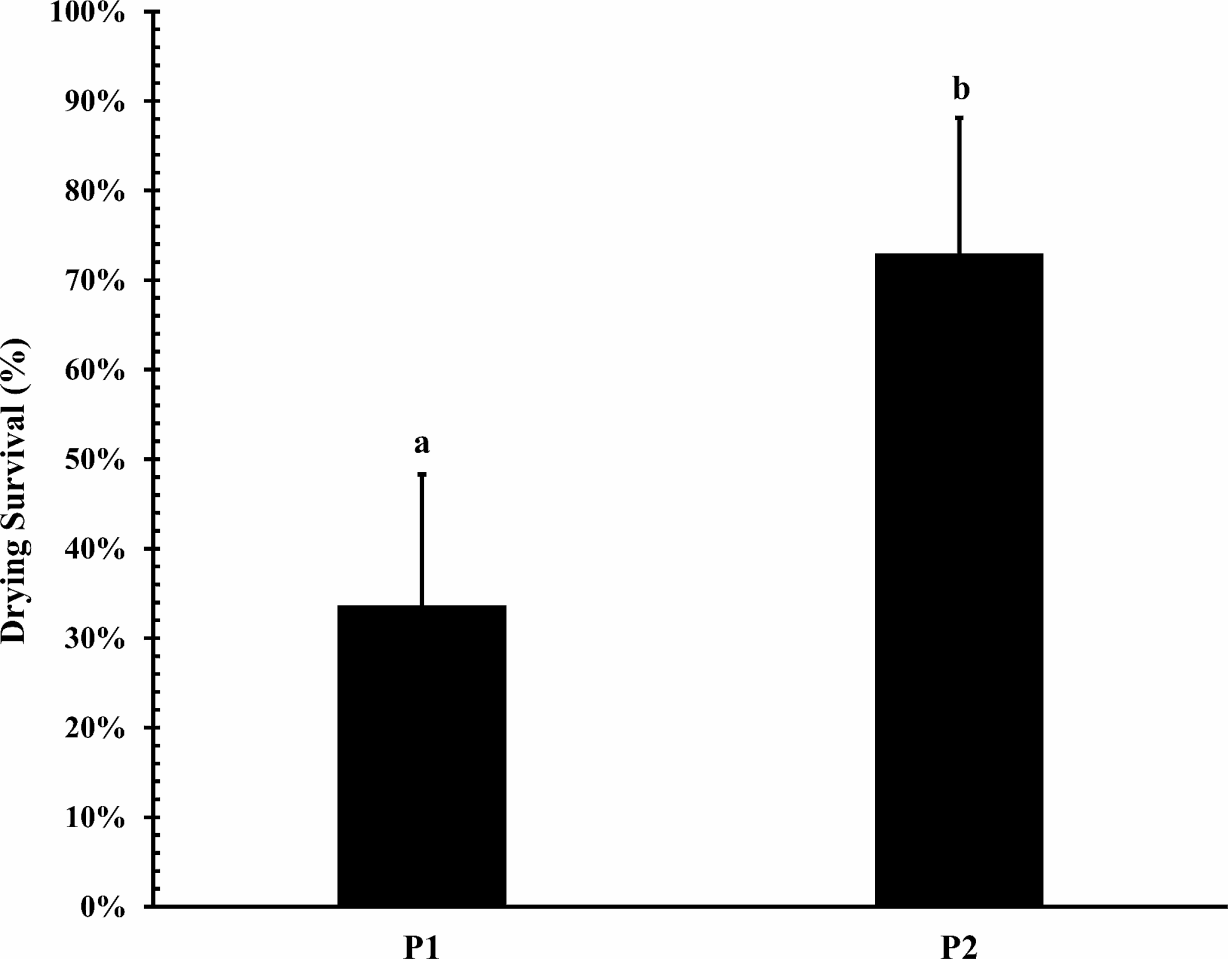
Drying survival of immobilized *T. koningiopsis* Th003 conidia in the prototypes of hydrogel capsules P1 and P2 (mean ± SD). Treatments with different letter are significantly different according to the Tukey’s honestly significant difference (HSD) procedure

P2 prototype provided to *T. koningiopsis* Th003 higher desiccation tolerance than P1 prototype. Survival of Th003 conidia was significantly lower in the P1 prototype with 33.66% ± 14.6% (F_1, 14_ = 24.75; *P* = 0.0003) compared to P2 with 72.97 ± 15.5%. Interestingly, Th003 performed relatively high drying survival, hence viability losses of immobilized conidia were less than 1 log unit (Fig. 1).

### Efficacy of encapsulated *T. Koningiopsis* Th003 against rice sheath blight

**Fig. 2:**
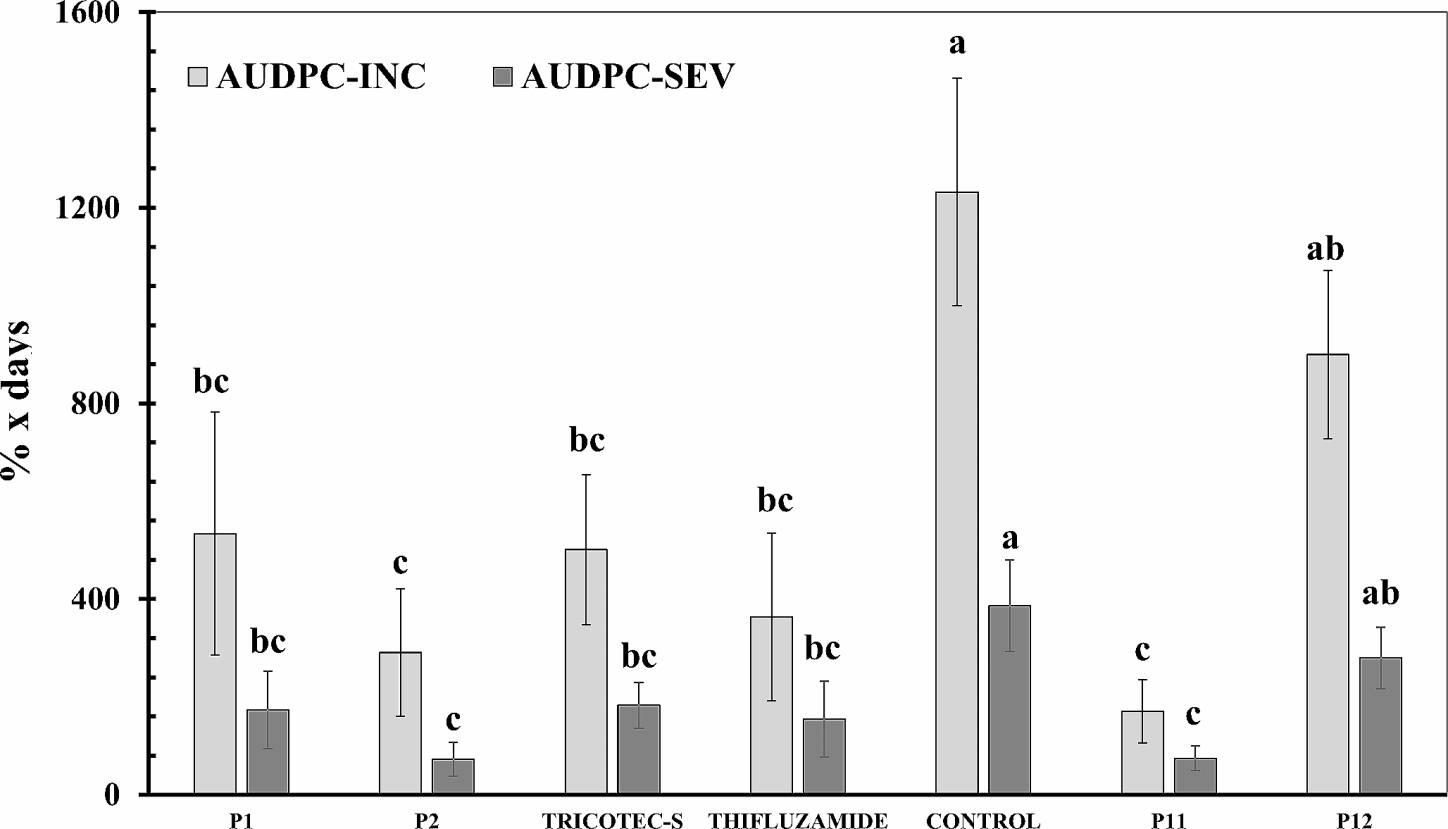
Area under incidence and severity progress curves (AUDPC-INC and AUDPC-SEV, respectively) of rice sheath-blight disease in response to the application of new prototypes of microcapsules containing conidia of *T. koningiopsis* Th003 (P1 and P2). These treatments were compared to the commercial biofungicide TRICOTEC® WG (1 g L^-1^ = 1 × 10^6^ conidia mL^− 1^ of Th003 and 50 mL per pot) applied once at sowing of rice seeds (TRICOTEC-S); the chemical fungicide THIFLUZAMIDE (1,5 mL L^− 1^) sprayed at 30 and 45 days after seedlings emergence; control of capsules without Th003-conidia, containing only formulation carriers (P11 and P12, respectively); and to the rice plants grown without treatments against the disease (CONTROL). Columns with the same letter are not significantly different according to Duncan’s multiple range test for each variable (α = 0.05). Bars on the top of the columns represent the standard error of the data (*n* = 6). AUDPC-INC and AUDPC-SEV were separately compared

It was observed that rice plants treated with the commercial biofungicide TRICOTEC®, when strictly following the manufacturer’s instructions (TRICOTEC-EC), were almost free of the disease, since just two experimental units showed symptoms in 20% and 22% of the plants at 49 and 69 days after planting, respectively. Thus, this treatment was not included in the statistical analysis, but TRICOTEC applied only at planting time (TRICOTEC-S) was included as the accurate biological treatment to compare the effect of capsules prototypes. The initial symptoms of the sheath blight disease were observed on rice seedlings at 14 days after planting in all treatments, except for the prototype-control P11 in which the expression of the disease symptoms was delayed until 49 days after planting (Table 2). The ANOVA showed a significant effect of the treatments on the initial level of the disease incidence (F_6,35_ = 4.71; *P* = 0.0018) and severity (F_6,35_ = 3.46; *P* = 0.0106), which were significantly higher in the experimental units of the negative control than those treated against the disease. However, no significant effects of the treatments on the final level of the incidence (F_6,35_ = 2.19; *P* = 0.0732) and severity (F_6,35_ = 1.86; *P* = 0.1223) were detected by the ANOVA (Table 2). The analysis of the effect of treatments on the AUDPC showed that prototype-control P12 containing only carriers was not significantly different compared to the negative control, while all other treatments showed significant differences, both on AUDPC-INC (F_6,35_ = 4.49; *P* = 0.0023) and AUDPC-SEV (F_6,35_ = 3.33; *P* = 0.0124) variables. However, no significant differences were observed among these treatments (Fig. 2). In particular, the symptoms of *R. solani* such as rusty-brown and dry sunken lesions on roots appeared on negative control, but those were not observed in prototypes treatments (P1 and P2) (Fig. 2S). While the efficacy to reduce the disease incidence in plants treated with the P11 control-prototype, and the fungicide Thifluzamide was 82% and 77% on average respectively, the rest of the treatments showed an efficacy from 23% to 61%, but no significant differences were detected among the efficacy of the treatments (F_5,30_ = 2.03; *P*= 0.0109) (Table 2).

**Table 2:** Response of rice sheath-blight disease variables to various treatments based on biological formulations

Treatment ^a^	Period of Incubation (days) ^b^	Initial incidence (%) ^c^	Initial severity (%) ^c^	Final incidence (%) ^d^	Final severity (%) ^d^	Efficacy on AUDPC-INC (%) ^e^	Efficacy on AUDPC-SEV (%) ^e^
P1	14	3.70 (2.34)^f^ bc	0.82 (0.52) bc	12.63 (5.71) ab	7.33 (3.31) ab	60.77 (18.22) ab	59.41 (19.53) ab
P2	14	3.33 (2.11) bc	0.55 (0.38) bc	5.42 (2.45) b	2.87 (1.45) b	67.23 (16.58) ab	68.69 (15.99) ab
TRICOTEC-S	14	3.52 (2.23) bc	1.19 (0.83) bc	12.27 (3.04) ab	6.75 (1.95) ab	51.06 (15.96) b	39.09 (15.22) b
THIFLUZAMIDE	14	1.85 (1.85) c	0.41 (0.41) c	11.11 (5.74) b	5.35 (3.07) b	77.06 (10.57) a	71.69 (12.92) a
CONTROL	14	16.94 (3.73) a	3.33 (1.13) a	22.78 (4.39) a	11.75 (2.36) a	-	-
P11	49	0.00 (0.00) c	0.00 (0.00) c	10.08 (2.57) ab	5.73 (2.08) ab	81.60 (9.25) a	66.82 (15.63) ab
P12	14	9.60 (3.50) b	1.74 (0.79) b	23.97 (6.49) a	11.33 (3.09) a	23.46 (11.16) b	24.11 (11.88) b

^**a**^ Rice plants were grown in soil artificially inoculated with *R. solani* Rh002; P1 and P2 = microencapsulate prototypes based on *T. koningiopsis* Th003 applied once at sowing of rice seeds; TRICOTEC-S = commercial biofungicide TRICOTEC WG based on Th003, applied by drench once at sowing; THIFLUZAMIDE = chemical fungicide Pulsor 2SC (SummitAgro) at dose of 1,5 mL L-1 sprayed at 30 and 45 days after seedlings emergence according to the manufacturer directions; CONTROL: untreated plants; P11 and P12 = microencapsulate prototypes free of Th003. ^**b**^ Days from sown until symptoms expression of disease in rice plants.^**c**^ Disease incidence and severity at 14 days after planting and ^d^ Disease incidence and severity at 69 days after planting. Values represent the average of 6 replications; analyses were performed after arcsine-square root transformation of the corresponding percent data.^**e**^ The efficacy of treatments for reduce the area under incidence and severity progress curves (AUDPC-INC and AUDPC-SEV, respectively) was calculated with respect to the CONTROL.^**f**^ Value in parentheses represent the standard error of six replications from two independent experiments with similar results

Treatments in each column with the same letter are not significantly different according to Duncan’s multiple range test (α = 0.05)

### Effect of pH on releasing of encapsulated conidia

**Fig. 3:**
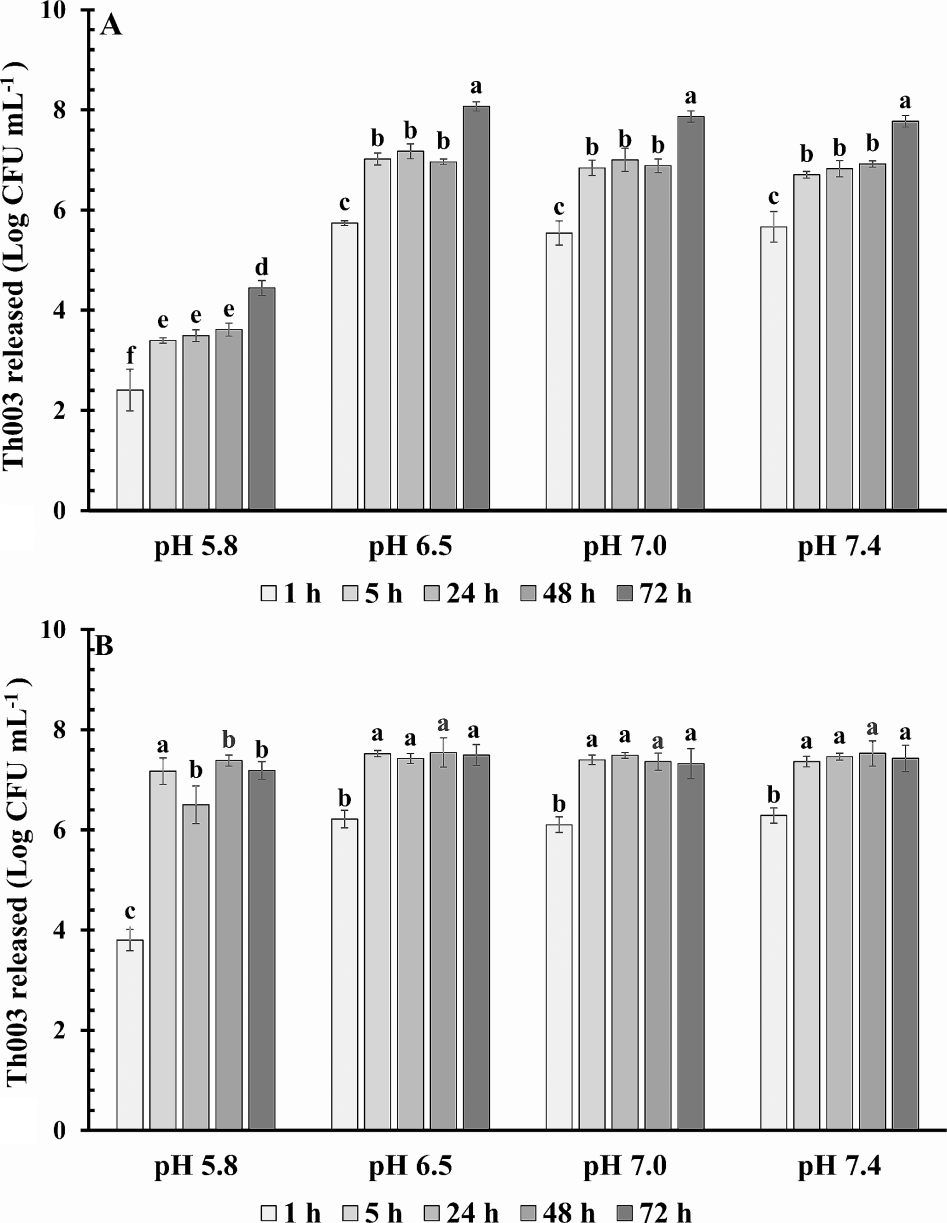
Conidia release profiles of *T. koningiopsis* Th003 in capsule prototypes under different pH values **A**: P1; **B**: P2. Treatments with the same letter are not significantly different according to the Tukey’s honestly significant difference (HSD) procedure at *p* < 0.05 confidence level

The capsule prototype components exerted a significant effect on the conidia release (F_1,119_ = 356.30; *P* < 0.0000), the pH also showed a significant effect (F_3,119_ = 405.45; *P* < 0.0000) as well as the time (F_4,119_ = 167.13; *P* < 0.0000). The release of conidia from prototypes P1 (F_3,59_ = 1479.91; *P* < 0.0000) and P2 (F_4,59_ = 251.25; *P* < 0.0000) was significantly affected at pH 5.8 after 1 h (Fig. 3A and B). Nevertheless, it was observed that the number of released conidia was lower in P1 than in P2 under pH 5.8 throughout the evaluation time. In terms of time, the released conidia at 1 and 72 h were significantly different in P1 (F_4,59_ = 251.24; *P* < 0.0000), but no significant differences were detected for released conidia between 5 and 48 h. Whereas with prototype 2 between hours 5 and 72, the number of conidia releases no evidence significative differences in the time, but until 1 h it was significantly different (F_4,59_ = 202.43; *P* < 0.0000) (Fig. 3A and B). P2 showed more homogeneous conidia release than P1, since 5 h until 72 h the evaluated time and a pH between 6.5 and 7.4 a significant effect of pH on conidial release was observed in P1 compared to prototype P2 (F_4,59_ = 251.24; *P* < 0.0000). The number of conidia released from P1 at pH 5.8 was significantly lower compared to values observed at pH levels of 6.5, 7.0, and 7.4 at all evaluated time points (F_4,59_ = 202.43; *P* < 0.0000). Additionally, it was evidenced that the number of conidia released from P1 was lower after 1 h of evaluation, while remaining constant from 5 to 48 h, significantly increasing only at 72 h of incubation (Fig. 3A). This outcome suggests a controlled conidial release, as the number remained constant between 5 and 48 h and only increased after 72 h of incubation. Regarding P2, a lesser negative effect of pH on conidial release was observed, as the number of released conidia exhibited a similar behavior across different pH levels. Moreover, it was observed that the majority of conidia were released after 5 h of incubation, maintaining this value up to 72 h (Fig. 3B).

### Particle size

**Fig. 4:**
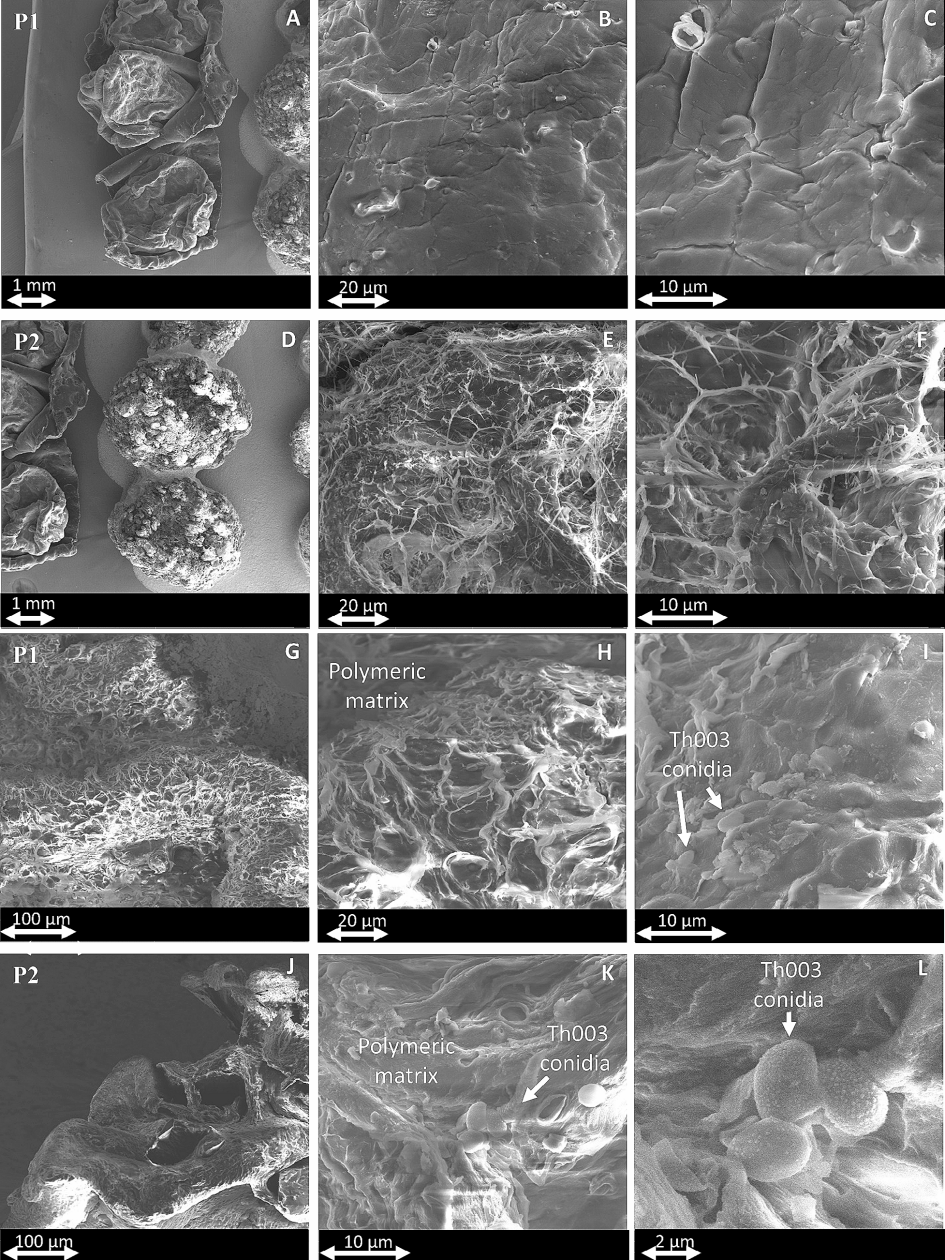
SEM micrographs of dried prototypes P1 and P2 focusing on surface topography (P1: A, B, C; P2: D, E, F) and inner structures (P1: G, H, I; P2: J, K, L)

The shape of the capsules for prototypes P1 and P2 was spherical and the size was homogeneous under both the wet and dry state after the fluid bed operation (Fig. 1S). The diameter of the capsules in prototype P1 was found within a minimum range of 670 to 1013 μm; being smaller than those of the P2 prototype, which were in the range of 1430 to 2270 μm (Fig. 4SA). The rate of capsule sphericity (φ) was 0.0008 to 0.2742 and 0.000 to 0.2789 for P1 and P2, respectively. Additionally, most of the P1 capsules (> 50%) had a particle size of 768 to 867 μm, with an average diameter of 820 μm and 0.0974 (φ), while > 70% of the P2 capsules had a size in the range of 1550 to 1990 μm with an average diameter of 1775.1 μm and 0,8343 (φ) (Fig. 4SB).

### Scanning electron microscopy (SEM)

The micrographs display the surface features of the fluid bed-dried P1 and P2 capsules. Prior to drying, these capsules possessed a regular, spherical shape, were mononuclear, opaque, and showcased a dark coloration, attributed to the inclusion of biochar (Fig. 4). Irregular and wrinkled surfaces were present in all dry capsules, as well as shrinkage due to dehydration. In general, it can be observed that the alginate-based prototype (P1) tended to have a smoother surface at maximum magnification (6000x) (Fig. 4A, B, C), while with amidated pectin-based (P2) a fragmented and irregular surface predominated (Fig. 4D, E, F). Conidia were not observed on the dry surface of the capsules, indicating that the cells and structures of the fungus were completely inside the polymeric matrix. The SEM photomicrographs show the interior of the prototypes P1 (Fig. 4G, H, I) and P2 (Fig. 4J, K, L). The presence of the immobilized Th003 conidia was confirmed in both polymeric matrices. Most of the observed conidia were independently trapped in the biopolymer matrix.

### Stability of prototypes under storage

The prototype P1 showed a greater loss of viability (6.28%) compared with the prototype P2 (2.51%) at 6 months after storage under 8 °C. Both prototypes showed a significantly higher loss of viability (> 15%) at 18 °C than the loss to 8 °C (Table 3). Prototype P2 showed a significantly higher loss of viability (> 50%) compared to prototype P1 under 28 °C (Table 3).

**Table 3:** Viability loss (%) and shelf-life estimation of *T. koningiopsis* Th003 prototypes (P1, P2). P1 and P2 prototypes were stored for six months at 8 ± 2 °C, 18 ± 2 °C and 28 ± 2 °C. * For viability loss: different letters represent significant differences according to Tukey´s multiple comparisons test (α = 0.05). SD: standard deviation; *n* = 3 independent replicates

Prototype	Storage temperature (°C)	Viability loss (%) (mean ± SD) *	Shelf life (months)
P1	**8 °C**	6.28 ± 0.01 c	8.79
	**18 °C**	16.78 ± 0.03 b	6.53
	**28 °C**	18.64 ± 0.09 b	6.28
P2	**8 °C**	2.51 ± 0.07 a	8.11
	**18 °C**	17.60 ± 0.04 b	6.76
	**28 °C**	53.29 ± 0.04 c	2.53

The viability (CFU/capsule) was correlated with storage time using the polynomial approach. Thus, the shelf-life was estimated using the generated equation and is presented in the Table 2S. In general, the shelf life was inversely proportional to the temperature. The mathematical model predicted that the prototypes P1 and P2 stored at 8 °C could maintain its viability for around 8 months.

## Discussion

The incorporation of biocontrol agents in the agroecosystems through the direct application to the soil, the root immersion in microbial suspension, or the foliar spraying are not economically practical at large scale, due to the high amount of inoculum that can be required. Conversely, the application of capsules provides an easy and sometimes unique application, an easy storage and transport (dried formula) (Locatelli et al. 2018). Encapsulation presents advantages for biocontrol agents in the field, protecting them from biotic and abiotic effects, provides higher stability and can promotes the effectiveness for controlling plant pathogens, besides desirable characteristics from capsules components like biocompatibility and biodegradation in soil (Maruyama et al. 2020). For instance, it has been found that encapsulation of *T. harzianum* improved the chitinolytic and cellulolytic activity and biocontrol of *S. sclerotiorum* compared to the non-encapsulated fungus (Maruyama et al. 2020). Thereby, this type of formulation can improve the survival of the biocontrol agent in the soil and provide less exposition to external factors (UV, temperature, pH, etc.).

In this work, it was found that the development of the sheath-blight disease in rice plants treated with the prototypes P1 or P2, was similar to one application of the commercial bioproduct TRICOTEC® WG based on *T. koningiopsis* Th003, suggesting that the new formulation of the fungus did not affect the biocontrol activity of Th003. The final values of the incidence and severity of the disease was significantly lower only in plants treated with the prototype P2 and Thifluzamide as compared to the control, indicating a better behaviour than P1. Interestingly, the carrier P11 showed some important control activity of the disease, although it was not statistically different compared to the control. However, the effect of both carriers was demonstrated as indirect on *R. solani*, since there was no inhibition of the growth of Rh002 under an in vitro antagonism test with the carriers P11 and P12 (Fig. 3S), possibly activating the defence mechanisms in the plant.

Alginate, which was one of the compounds in P1 and P11 prototypes, induced resistance against *Pseudomonas syringae* pv. *tomato* via salicylic acid in *Arabidopsis thaliana* (Zhang et al. 2019). Alginate has been used as an elicitor to control *Alternaria solani* in tomato (Dey et al. 2019). Alginate-deriving oligosaccharides have been reported as elicitors stimulating the accumulation of phytoalexin and inducing phenylalanine ammonia lyase in soybean and antimicrobial activity against *Pseudomonas aeruginosa* (An et al. 2009). Conversely, the carrier that contained amidated pectin did not show a reduction in the progress of the disease and neither in vitro antagonism assay. Since the carrier P12 did not show reduction of sheath blight progress, here the efficacy of the treatment P2 clearly it is attributed to Th003.

Both prototypes P1 and P2 contained biochar, which is an amendment reported as an inductor of resistance in several plants, depending on the type of biochar feedstock and application rate (Copley et al. 2017). Biochar has been evaluated in cucumber, showing positive results at low concentrations against *R. solani* (Jaiswal et al. 2014). In rice plants, it can improve the grain yield (Asai et al. 2009) and tolerance to cold stress (Yuan et al. 2017). The other shared compound in these capsules was polydextrose, a well-recognized drying protector. Frequently, it is used during dehydration process for protecting different cells such as *Lactobacillus acidophilus* (Okuro et al. 2013), *Lactobacillus rhamnosus* (Ananta et al. 2005) and *Trichoderma* sp. (Cortés et al. 2021). Besides, polydextrose within Th003 conidia membrane may form a viscous layer, preventing growth of ice crystals and preserving the cell structure (Hubálek 2003).

The damage caused by cell dehydration is a critical factor to consider during the process of formulating microorganisms as active ingredients of bioproducts (Faria et al. 2017). Generally, the drying processes used for biological formulations involve both deleterious effects, so drying strategies to avoid stress are urgent. Here, the prototypes P1 and P2 of hydrogel capsules based on alginate and amidated pectin biopolymers, respectively, were proposed. Pectin have provided desiccation tolerance to several types of cells, including conidia of *Trichoderma* strains (Cortés et al. 2021). Similarly, dried alginate-based capsules mitigated dehydration stress on cells due to resistant membranes, and they dried essentially as a water droplet (Pereda et al. 2019). These properties could contribute to the microbial survival within the capsules by creating a microenvironment that isolates the conidia from water vapor or reactive oxygen agents.

Calcium gluconate was used as an advantageous cross-linker in both prototypes (P1, P2), since this compound provides protective effects to microorganisms in the dehydration processes, when it is used as a crosslinking agent compared to calcium chloride (Schoebitz et al. 2012). These effects of greater survival have been evidenced in conidia of *M. brunneum* and *S. cerevisiae* (Humbert et al. 2017). Crosslinking agents, can have protective drying effects due to their non-cationic structure, they accumulate intracellularly and serve as compatible solutes, lodging in the membrane and modifying the glass transition temperature. They also slow the diffusion processes and can increase the hygroscopicity of the capsules, the source of nutrients for fungal cells (Humbert et al. 2017).

Considering that pH affects the degradation of polymers and their hydration capacity, it is likely that, at pH 5.8, the high concentration of hydrogen ions (H +) increased the stability of the gel’s crosslinking, making it more insoluble (Wang et al. 2023; Wong et al. 2011). When dissolving the prototype P1 in the buffer adjusted to pH 5.8, it was observed that they took on a more consistent gel-like appearance, and their size increased. This can be attributed to the fact that the carboxylic groups of the organic acids present accept protons, increasing the formation of bonds with glucuronic acid (Block G) in alginate. The properties like pore space, release rate, density and pressure of the capsules depend on their size and shape (Zhao and Chew 2020). As the capsules of this study have an inner liquid core (Th003 conidia suspension) and a solid shell, the evaluation of the particle size is an important characteristic because of its influence on the viability of the conidia and release rate. It is probable that the smaller capsules have the more efficient diffusion of O_2_, nutrients, and metabolites, increasing concentration and viability. In contrast, the larger capsules have a limited oxygen diffusion rate in the center of the capsule, thus affecting the viability of the conidia (Kopač et al. 2023; Lee et al. 2014).

The stability of conidia against stress factors such as temperature and storage are a desirable attribute in biopesticides. The encapsulation technique is one of the most studied strategies to improve the survival and biological activity of microorganisms (Mancera-López et al. 2019; Muñoz-Celaya et al. 2012) and according to Maruyama et al. (2020), this type of formulation have a variable range of shelf life between 3 and 10 months at refrigeration temperature (4 °C). In this work, the viability of conidia during storage was higher in samples at 8 °C than 18 °C and 28 °C, probably due to the higher reactivity and diffusivity of reactive oxygen species, or to an increase of their metabolic activity (Muñoz-Celaya et al. 2012). These results suggest that the biopolymer matrices delayed the diffusion of oxygen into the microcapsules at 8 °C, limiting the amount of available oxygen for participating in the oxidation of macro-molecules and causing oxidative stress. In this work, the prototypes P1 and P2 have storage stabilities according to this type of formulation with values around 8 months at 4 °C. However, P1 stored at 18 °C and 28 °C showed a shelf life of 6 months and can be an alternative to storing this prototype at room temperature, an attractive and cost-efficient option.

Although encapsulation as a formulation strategy has shown great potential, most of the reported investigations have been at the laboratory level and few commercial encapsulated products are available. However, lower production costs and advanced technical handling developed with techniques like the *Jet-Cutter* would improve the adoption (Preibisch et al. 2018; Yerramathi et al. 2023). New approaches must remain affordable and compatible with current application technologies that provide flexibility to farmers.

To conclude, this study demonstrated that amidated pectin within the encapsulating matrix provides higher drying survival of *T. koningiopsis* Th003 and advantages on conidia releasing upon acidic conditions at pH 5.8 compared to alginate. Conversely, for extending storage stability, alginate is a better alternative specially at high temperatures, such as 28 °C. Both biopolymers facilitate the antagonistic activity of Th003 against *R. solani*, and therefore can be incorporated in hydrogel capsules for novel bioinoculants development.

### Electronic supplementary material

Below is the link to the electronic supplementary material.

Supplementary Material 1

## Data Availability

All datasets generated for this study can be provided upon request.
