# Supplementary material for: Hydrogel capsules as new delivery system for Trichoderma koningiopsis Th003 to control Rhizoctonia solani in rice (Oryza sativa)
Source: World J Microbiol Biotechnol. 2024 Feb 26;40(4):108. doi: 10.1007/s11274-024-03897-0 (PMC10894772; doi:10.1007/s11274-024-03897-0)
Supplement: Supplementary file 1 — Supplementary Material 1 [file 11274_2024_3897_MOESM1_ESM.docx]

**Hydrogel capsules as new delivery system for *Trichoderma koningiopsis* Th003 to control *Rhizoctonia solani* in rice (*Oryza sativa*)**

**SUPPLEMENTARY MATERIAL**

**Fig. 1S.** Prototypes of hydrogel capsules P1 and P2 before (Wet) and after fluid bed drying operation (Dry)


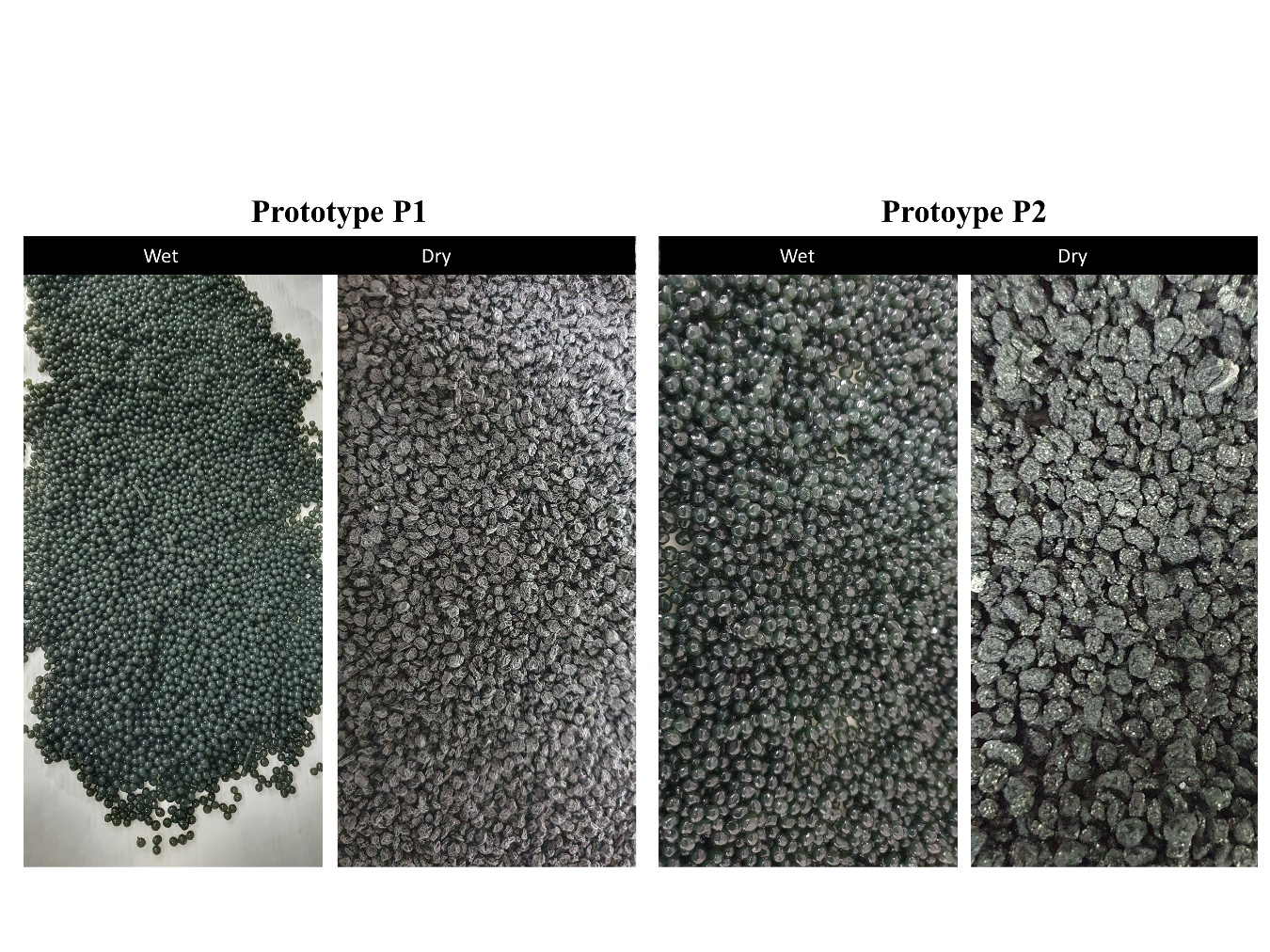


**Table 1S.** Scale of sheath blight disease severity in rice plants

| Level | Symptoms description |
| --- | --- |
| 0 | Healthy plants, no symptoms of the disease are observed. |
| 1 | Dark brown oval lesions restricted to the level of the water line or site of infection. |
| 2 | Few oval lesions or joints with wide edges on the lower leaf sheath or at the sites of infection, 5% of tissue affected. |
| 3 | Lesions on the lower sheath of the leaf or at the points of infection, joint lesions, less than 10% of the affected tissue. |
| 4 | Lesions restricted mainly to the sheath on the lower third of the plant, low leaves or other infection points, discrete lesions or joints with narrow red-brown borders, 10 to 15% of the leaf and envelope tissue affected. |
| 5 | Lesions mainly restricted to the sheath and leaves of the lower half of the plant, lesions usually close together with long necrotic centers and narrow red-brown borders, 15 to 25% of affected tissue. |
| 6 | Lesions usually together and affecting the lower two-thirds of the area of the plant sheath, lesions extended to the lower part of the leaf or lower leaves dead due to injury to the sheath, 25 to 40% of tissues affected. |
| 7 | Lesions usually together and affecting three-quarters of the area of the plant sheath, lesions extended to the leaves of two-thirds of the plant, 40 to 60% of affected tissue. |
| 8 | Lesions reaching the flag leaf, low sheath with joint lesions covering most of the tissue, lower and middle leaves dead, 60 to 80% of tissues affected. |
| 9 | Lesions reaching the flag leaf, most of the lower leaves dead, dry sheath, brown stem, collapse, 80 to 100% of affected tissue. |

**Table 2S.** Mathematical models used to correlate viability *vs.* storage time and corresponding squared correlation coefficients (R2) and polynomial model equation.

| **Prototype** | **Storage temperature (ºC)** | **Mathematical models (R2)** | | | | | | **Equation** |
| --- | --- | --- | --- | --- | --- | --- | --- | --- |
|  |  | **Zero orden** | **First orden** | **Higuchi** | **Kosmeyer pepar** | **Hisson- Crowel** | **Polynomial** |  |
| **P1** | **8°C** | 0.3721 | 0.3708 | 0.3363 | 0.3465 | 0.3714 | 0.6260 | $y= 0.0261\mathcal{x}^{3}- 0.2152\mathcal{x}^{2} + 0.2648\mathcal{x}+ 6.4594$ |
|  | **18°C** | 0.5920 | 0.5781 | 0.6390 | 0.6369 | 0.5829 | 0.9861 | $y= 0.0246\mathcal{x}^{3}- 0.1693\mathcal{x}^{2} + 0.0192\mathcal{x}+ 6.5121$ |
|  | **28°C** | 0.4897 | 0.4795 | 0.6727 | 0.5965 | 0.4831 | 0.8718 | $y= 0.0689\mathcal{x}^{2}- 0.5923\mathcal{x}+ 6.5873$ |
| **P2** | **8°C** | 0.1241 | 0.1136 | 0.2391 | 0.0739 | 0.1170 | 0.7995 | $y= -0.0137\mathcal{x}^{3}+ 0.1429\mathcal{x}^{2}- 0.3877\mathcal{x}+ 6.6576$ |
|  | **18°C** | 0.4910 | 0.4888 | 0.6569 | 0.4514 | 0.4896 | 0.8592 | $y=0.027\mathcal{x}^{2}- 0.3734\mathcal{x}+ 6.7209$ |
|  | **28°C** | 0.7517 | 0.7560 | 0.8221 | 0.8735 | 0.7549 | 0.8897 | $y=0.0973\mathcal{x}^{2}-1.2616\mathcal{x}+ 7.0708$ |

**Fig. 2S.** Rice plant appearance at 69 days after the application of the capsule prototypes. The symptoms of the disease consisted of greenish-gray elliptical or ovoid lesions on the sheaths and leaves, reduced root volume and brown coloration of the roots.

**
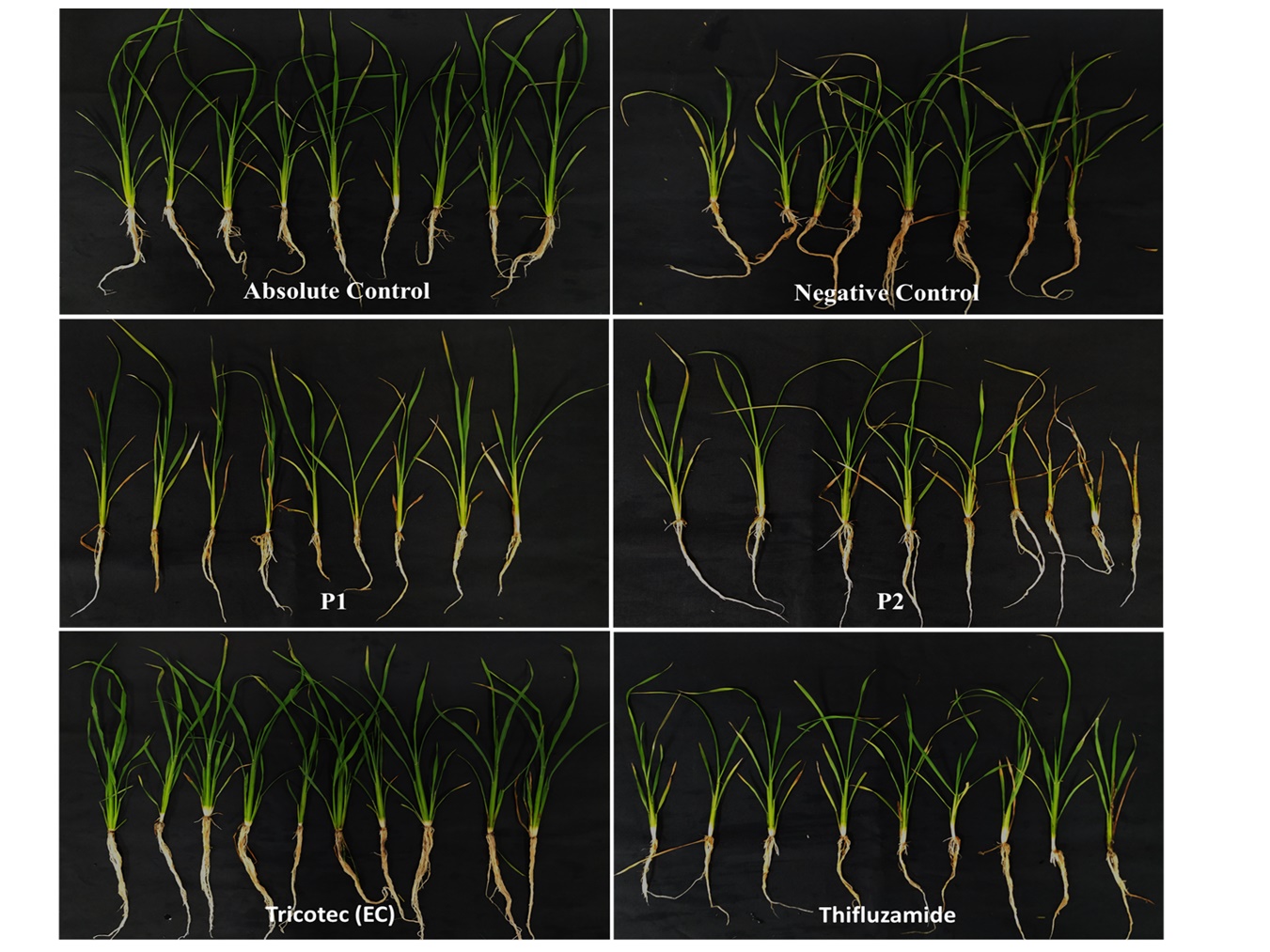
**

**Antagonistic test under *in vitro* conditions**

With the aim to verify the biological activity of the capsules prototypes, both those based on Th003 and those free of the biocontrol agent (carriers), an antagonistic test was carried out in Petri dishes. A square plug (0.5 cm) from a 10-day old PDA culture of *R. solani* Rh002 was placed in the center of a Petri dish with PDA medium. Then, two capsules were placed 2 cm apart from the edge of the plate, on two opposite sides. Capsule prototypes containing only the carriers (P11 and P12) were included as controls, conidia of Th003 was included as positive control, and Petri dishes inoculated only with *R. solani* was included as negative control. The inoculated dishes were incubated for seven days under 28 °C and darkness. Then, the diameter of *R. solani* colony was measured and the inhibition of growth by the treatments was calculated. The experiment had five dishes per treatment and was performed three times.

**Fig. 3S.** *In vitro* antagonism assay of prototypes and carriers against *R. solani* Rh002, seven days after inoculation. P1 showed 100 % of inhibition while P2 showed 96% in average. Carriers did not show inhibition of *R. solani* growth.


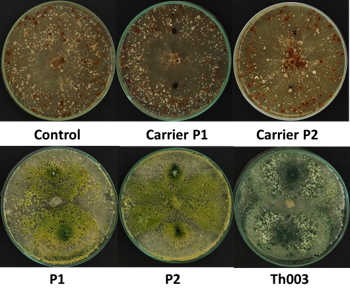


**Fig. 4S.** Particle size distribution for dry hydrogel capsules. **A**. P1 (Alginate) and **B**. P2 (Amidated Pectin).

**
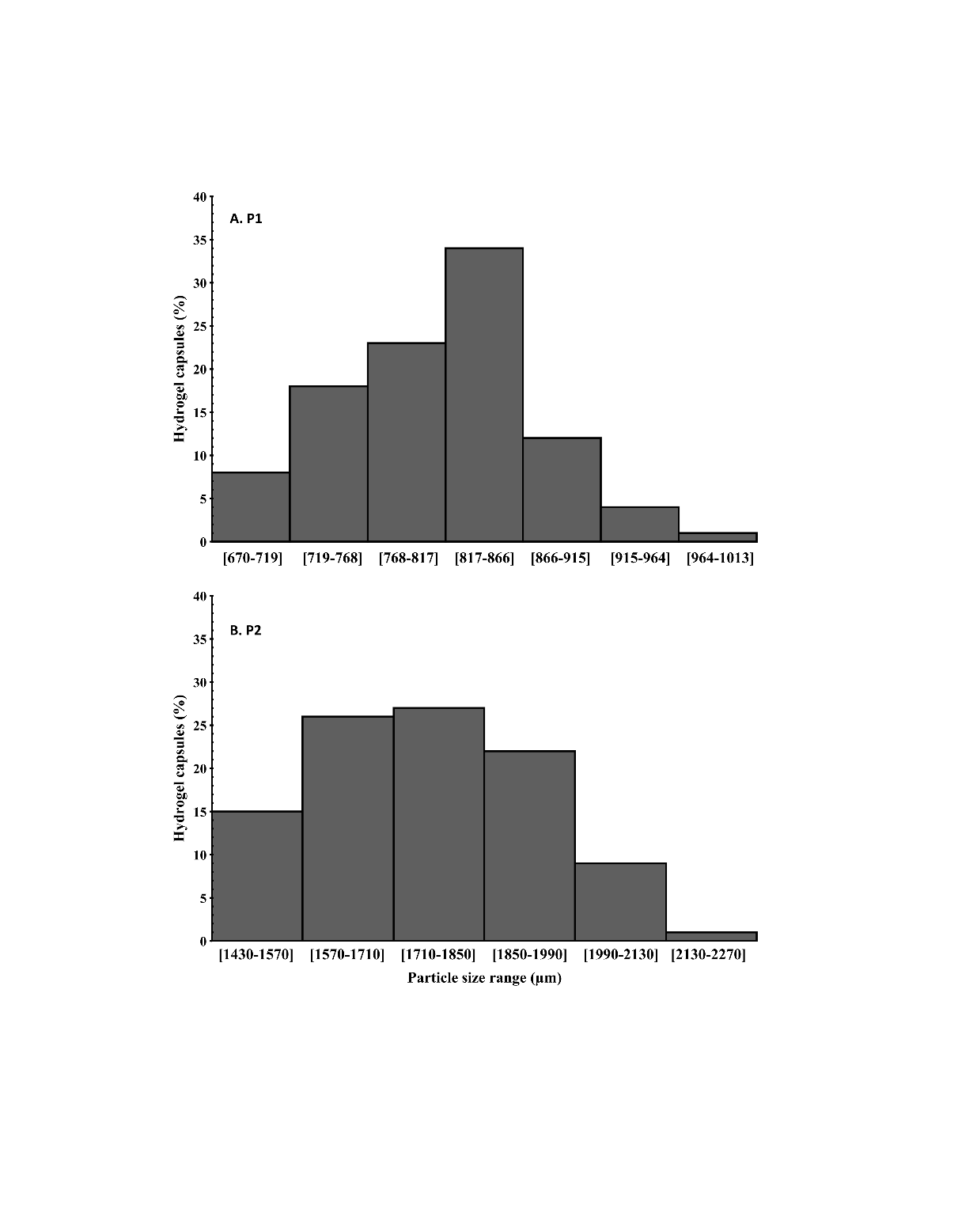
**
